# Rab33a and Rab33ba mediate the outgrowth of forebrain commissural axons in the zebrafish brain

**DOI:** 10.1038/s41598-018-38468-5

**Published:** 2019-02-12

**Authors:** Liguo Huang, Akihiro Urasaki, Naoyuki Inagaki

**Affiliations:** Laboratory of Systems Neurobiology and Medicine, Division of Biological Science, Nara Institute of Science and Technology, Ikoma, Nara 630-0192 Japan

## Abstract

Rab small GTPases play key roles in intracellular membrane trafficking. Rab33a promotes axon outgrowth of cultured rat hippocampal neurons by mediating the anterograde axonal transport of Golgi-derived vesicles and the concomitant exocytosis of these vesicles at the growth cone. However, the functions of Rab33 *in vivo* are unclear. Here, we show that zebrafish *rab33a* and *rab33ba* are orthologs of mammalian *Rab33a* and *Rab33b*, respectively. They are expressed in the developing brain, including in neurons of the telencephalic dorsorostral cluster and the diencephalic ventrorostral cluster, which project axons to form the anterior and postoptic commissures, respectively. Although *rab33a* single mutant and *rab33ba* single mutant fish did not show remarkable defects, fish carrying the *rab33a*;*rab33ba* double mutations displayed dysgenesis of the anterior and postoptic commissures. Single-cell labeling in the telencephalic dorsorostral cluster demonstrated that the *rab33a*;*rab33ba* double mutation inhibits axonal extension in the anterior commissure. These results suggest that Rab33a and Rab33ba mediate axon outgrowth and the formation of the forebrain commissures in the zebrafish brain in a cooperative manner.

## Introduction

Axon outgrowth requires rapid expansion of the plasma membrane^1^. Axonal membrane expansion is mediated by multiple processes, including membrane synthesis at the rough endoplasmic reticulum and Golgi apparatus in the cell body, vesicular transport along the axonal shaft, and vesicular exocytosis at the growth cone^1–5^. Rab family proteins are key regulators of intracellular vesicular trafficking pathways^6–10^. They localize to specific membrane compartments and function as molecular switches that cycle between the GTP-bound active form and the GDP-bound inactive form^6–10^. The GTP-bound active form recruits and activates specific effectors such as sorting adaptors, tethering factors, kinases, phosphatases and motor proteins, thereby regulating the specificity and directionality of vesicular trafficking^7–10^.

Rab33a^11^ is expressed in cells, including neurons, lymphocytes, melanocytes and parotid acinar cells^12–15^. In cultured rat hippocampal neurons, Rab33a is localized to the Golgi apparatus and post-Golgi vesicles transported along axons^14^. With regard to the mechanism for axonal membrane expansion, our previous study with cultured hippocampal neurons demonstrated that Rab33a promotes anterograde axonal transport of the post-Golgi vesicles, which is associated with vesicular exocytosis at the growth cones and axon outgrowth^14^. Rab33a is also reported to be involved in the regulation of vesicular exocytosis in parotid acinar cells, PC12 cells and umbilical vein epithelial cells^15–17^. In addition, Rab33a interacts with singar1/RUFY3^18^, which suppresses the formation of surplus axons in cultured hippocampal neurons^19^. However, the role of Rab33a in axon outgrowth *in vivo* remains unclear.

Rab33b is another Rab33 protein that is expressed ubiquitously in mouse tissues and is localized in the Golgi apparatus^20^. Rab33b interacts with Golgi proteins such as GM130, rabaptin-5 and rabex-5^21^, and modulates autophagosome formation by interacting with Atg16L^22^. In addition, mutations in human *RAB33B* are found in patients with an autosomal recessive skeletal dysplasia, Smith–McCort dysplasia^23–25^. In zebrafish, three *rab33* genes, *rab33a*, *rab33ba* and *rab33bb*, have been identified^26^. While *rab33a* and *rab33ba* are highly conserved in vertebrates, *rab33bb* is reported only in zebrafish.

In this study, we analyzed the functions of Rab33a and Rab33ba in the zebrafish forebrain, an easily accessible system for the analyses of axon tract formation *in vivo*^27–29^. We show that zebrafish *rab33a* and *rab33ba* are orthologs of mammalian *Rab33a and Rab33b*, respectively, and that *rab33a* and *rab33ba* mediate the outgrowth of forebrain commissural axons in the developing zebrafish brain.

## Results

### Zebrafish *rab33a* and *rab33ba* are orthologs of mammalian *Rab33a* and *Rab33b*, respectively

Zebrafish *rab33a*, *rab33ba* and *rab33bb* encode 236-, 239- and 227-amino acid (aa) proteins, respectively^26^ (Supplementary Fig. S1a). Zebrafish Rab33a has 74.9% identity with human RAB33A and 53.8% identity with human RAB33B, whereas zebrafish Rab33ba has 53.4% identity with human RAB33A and 66.3% identity with human RAB33B. On the other hand, zebrafish Rab33bb has 48.1% identity with human RAB33A and 47.3% identity with human RAB33B. Phylogenetic analysis revealed that zebrafish rab33 genes are clustered into separate groups (Supplementary Fig. S1b). Zebrafish *rab33a* and mammalian *Rab33a* belong to the same group, while zebrafish *rab33ba* and mammalian *Rab33b* belong to another group, which is distinct from the group of zebrafish *rab33bb* (Supplementary Fig. S1b).

Synteny analyses in human, mouse, chicken, *Xenopus*, zebrafish, medaka and fugu genomes showed that human, mouse, chicken and *Xenopus rab33b* share conserved synteny with zebrafish, medaka and fugu *rab33ba* (Supplementary Fig. S2c). In the case of *rab33a*, synteny was observed among human, mouse, chicken and *Xenopu*s, but disrupted in fish (zebrafish, medaka and fugu) (Supplementary Fig. S2a). We also observed disrupted synteny between *slc25a14* and *aifm1*, which flank human, mouse and chicken *rab33a*, in zebrafish (Supplementary Fig. S2b). On the other hand, we identified *rab33bb* only in zebrafish (Supplementary Fig. S2d). These data indicate that zebrafish *rab33ba* shares synteny with mammalian *rab33b* but not with mammalian *rab33a*. Together, the present data suggest that zebrafish *rab33a* and *rab33ba* are orthologs of mammalian *Rab33a* and *Rab33b*, respectively.

### Zebrafish *rab33a* and *rab33ba* are expressed in forebrain commissural neurons

**Figure 1:**
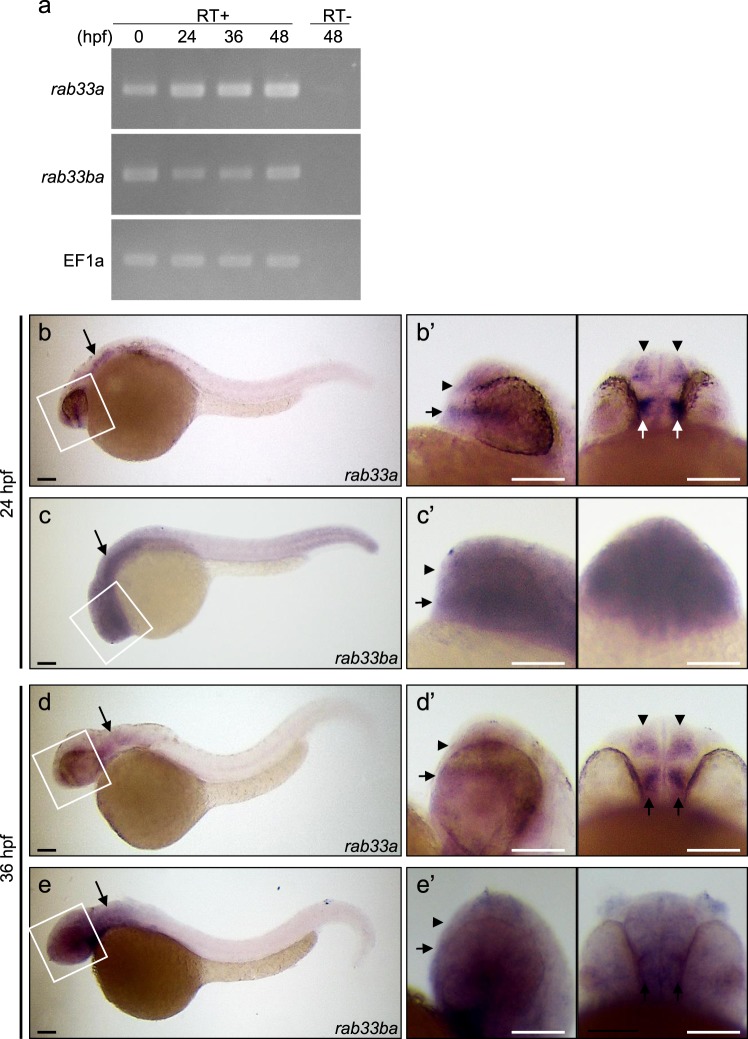
Expression of zebrafish *rab33a* and *rab33ba*. (**a**) RT-PCR analysis of *rab33a* and *rab33ba* transcripts. Elongation factor 1a (EF1a) was used as a control. Developmental stages are denoted as hours post-fertilization (hpf). RT-PCR products produced in the presence (RT+) or absence (RT−) of reverse transcriptase were electrophoresed on 2% agarose gels. (**b**,**c**) Whole-mount *in situ* hybridization of *rab33a* (**b**) and *rab33ba* (**c**) at 24 hpf; (b′) and (c′) show the enlarged lateral (left) and ventral (right) views of the areas indicated by the rectangles. (**d**,**e**) Whole-mount *in situ* hybridization of *rab33a* (**d**) and *rab33ba* (**e**) at 36 hpf; (d′) and (e′) show the enlarged lateral (left) and ventral (right) views of the areas indicated by the rectangles. Arrowheads and arrows in (b′–e′) indicate the DRC and VRC, respectively. Arrows in (**b–e**) indicate the hindbrain. See the negative control data for whole-mount *in situ* hybridization (Supplementary Fig. S3). Scale bars: 100 μm.

To examine the expression of *rab33a* and *rab33ba* in zebrafish embryos, we first performed RT-PCR using specific primers. RT-PCR analysis detected the expression of *rab33a* and *rab33ba* at 0, 24, 36 and 48 hours postfertilization (hpf) (Fig. 1a). Whole-mount *in situ* hybridization detected *rab33a* and *rab33ba* expression in the developing brain (Fig. 1b–e and Supplementary Fig. S3). At 24 hpf, *rab33a* was expressed in the forebrain, with high levels of the signal in the telencephalic dorsorostral cluster (DRC) (arrowheads, Fig. 1b′) and diencephalic ventrorostral cluster (VRC) (arrows, Fig. 1b′). These neuronal clusters are also referred as the nucleus of the tract of the anterior commissure and the nucleus of the tract of the postoptic commissure, respectively^30^. On the other hand, *rab33ba* was expressed widely in the forebrain including the DRC and VRC of 24 hpf embryos (arrowheads and arrows, Fig. 1c′). Neurons in the DRC and VRC start to extend axons at 20 hpf and form axonal tracts of the anterior commissure and postoptic commissure, respectively, by 36 hpf^27,28,30^. The expression of *rab33a* and *rab33ba* was also detected in the forebrain including the DRC and VRC of 36 hpf embryos (arrowheads and arrows, Fig. 1d′,e′), suggesting that these genes are expressed during axonal extension in the anterior and postoptic commissures. In addition, we detected the expression of *rab33a* and *rab33ba* in the hindbrain of 24 hpf and 36 hpf embryos (arrows, Fig. 1b–e).

### Rab33a and Rab33ba mediate the formation of forebrain commissures

**Figure 2:**
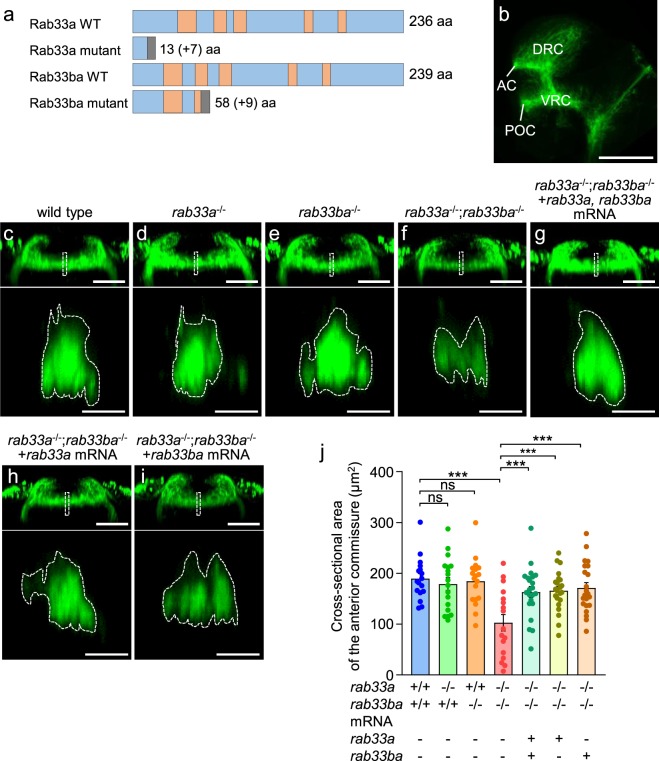
*rab33a*;*rab33ba* double mutants display a reduced cross-sectional area of the anterior commissure. (**a**) Schematic structures of Rab33a and Rab33ba proteins in wild-type and single mutant fish. Frameshift mutations in *rab33a* and *rab33ba* result in premature stop codons after aa positions 13 and 58, respectively. The gray boxes indicate amino acids added by the frameshift mutations, and the numbers in brackets indicate the numbers of these additional residues. The regions involved in GTP/GDP-binding and GTPase activity^11,17^ are indicated by the orange color. (**b**) A representative lateral view of a 36 hpf wild-type zebrafish brain immunolabeled with anti-acetylated tubulin antibody. See Movie 1. Abbreviations: AC, anterior commissure; POC, postoptic commissure. Scale bar: 100 μm. (**c–i**) Frontal views (upper panels) of the anterior commissures of wild-type control (**c**), *rab33a*^−/−^ single mutant (**d**), *rab33ba*^−/−^ single mutant (**e**) and *rab33a*^−/−^;*rab33ba*^−/−^double mutant (**f**) embryos at 36 hpf. In (**g**), *rab33a* and *rab33ba* mRNAs were injected into a *rab33a*^−/−^;*rab33ba*^−/−^ double mutant embryo for rescue analysis. In (**h**,**i**) *rab33a* mRNA (**h**) and *rab33ba* mRNA (**i**) were injected into a *rab33a*^−/−^;*rab33ba*^−^^*/*^^−^ double mutant embryo for rescue analysis. Scale bars: 50 µm. The lower panels show cross-sections at the dotted lines shown in the frontal views. Scale bars: 10 µm. (**j**) The cross-sectional area of the anterior commissure obtained from the data analyses in (**c–i**, lower panels). Wild-type control (n = 17), *rab33a*^−/−^ single mutant (n = 18), *rab33ba*^−/−^ single mutant (n = 18) and *rab33a*^−/−^;*rab33ba*^−/−^ double mutant (n = 17) embryos, and *rab33a*^−/−^;*rab33ba*^−/−^ double mutant embryos with *rab33a* and *rab33ba* mRNAs (n = 22), *rab33a* mRNA (n = 22) and *rab33ba* mRNA (n = 22) were analyzed at 36 hpf. Data are mean ± SEM; ^***^P < 0.01; ns, not significant (one-way ANOVA with Tukey’s post hoc test).

The expression of *rab33a* and *rab33ba* in the DRC and VRC suggest that Rab33a and Rab33ba may have a role in the formation of the anterior and postoptic commissures. To analyze the functions of *rab33a* and *rab33ba*, we generated *rab33a* and *rab33ba* mutants using the CRISPR/Cas9 system. To generate *rab33a* mutants, we injected *rab33a* gRNA and Cas9 mRNA into wild-type fertilized eggs. The injected fish were raised to adulthood and crossed with wild-type fish. The *rab33a* mutant allele contained a 10-bp deletion in the first exon, resulting in a frame shift and a premature stop codon after 13 aa (Fig. 2a and Supplementary Fig. S4a,b). To generate *rab33ba* mutants, we injected *rab33ba* gRNA and Cas9 mRNA into fertilized eggs from the *rab33a* heterozygous mutant fish. The injected fish were raised and crossed with wild-type fish. The *rab33ba* mutant allele harbored a 2-bp deletion (5-bp deletion and 3-bp insertion) in the first exon, resulting in a premature stop codon after 58 aa (Fig. 2a and Supplementary Fig. S5a,b). We identified homozygous and heterozygous fish for *rab33a* and *rab33ba* mutations by T7EI-based genotyping (Supplementary Figs S4c and S5c).

The DRC and VRC neurons start to develop axons at 24 hpf and form axonal tracts of the anterior and postoptic commissures by 36 hpf^27,30,31^. To visualize these axonal tracts, we performed whole-mount immunohistochemistry using anti-acetylated tubulin antibody^27,29,30^ (Fig. 2b). The anterior commissures in control and mutant embryos were analyzed at 36 hpf (Movie 1), and their cross-sectional areas were quantified at the midline (Fig. 2c–e). In control embryos, the cross-sectional area of the anterior commissure was 189.4 ± 10.1 μm^2^ (*n* = 17 embryos) (Fig. 2c,j), similar to that of the *rab33a* single mutant and the *rab33ba* single mutant embryos (Fig. 2d,e,j). We also generated a *rab33a*;*rab33ba* double mutant (Fig. 2f); the *rab33a*;*rab33ba* double mutation led to a significant reduction in the cross-sectional area of the anterior commissure (Fig. 2f,j). When *rab33a* and *rab33ba* mRNAs were injected into the double mutant embryos, the reduced cross-sectional area of the anterior commissure was rescued to a level similar to that of control embryos (Fig. 2g,j). Injection of *rab33a* mRNA or *rab33ba* mRNA into the double mutant also rescued the reduced cross-sectional area of the anterior commissure (Fig. 2h–j).

**Figure 3:**
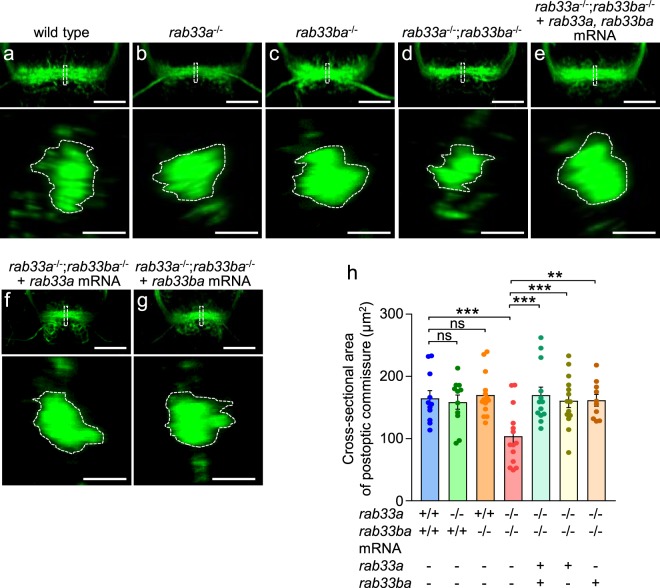
*rab33a*;*rab33ba* double mutants display a reduced cross-sectional area of the postoptic commissure. (**a–g**) Representative frontal views (upper panels) of the postoptic commissures of wild-type control (**a**), *rab33a*^−/−^ single mutant (**b**), *rab33ba*^−/−^ single mutant (**c**) and *rab33a*^−*/*−^;*rab33ba*^−*/*−^ double mutant (**d**) embryos at 36 hpf. In (**e**), *rab33a* and *rab33ba* mRNAs were injected into a *rab33a*^−*/*−^;*rab33ba*^−*/*−^ double mutant embryo for rescue analysis. In (**f**,**g**), *rab33a* mRNA (**f**) or *rab33ba* mRNA (**g**) were injected into a *rab33a*^−*/*−^;*rab33ba*^−*/*−^ double mutant embryo for rescue analysis. Scale bars: 50 µm. The lower panels show the cross-sections at the dotted lines shown in the frontal views. Scale bars: 10 µm. (**h**) The cross-sectional area of the postoptic commissure obtained from the data analyses in (**a–g**; lower panels). Wild-type control (n = 11), *rab33a*^−*/*−^ single mutant (n = 11), *rab33ba*^−*/*−^ single mutant (n = 14) and *rab33a*^−*/*−^;*rab33ba*^−*/*−^ double mutant (n = 14) embryos, and *rab33a*^−*/*−^;*rab33ba*^−*/*−^ double mutant embryos with *rab33a* and *rab33ba* mRNAs (n = 13), *rab33a* mRNA (n = 14) and *rab33ba* mRNA (n = 10) were analyzed at 36 hpf. Data are mean ± SEM; ^***^P < 0.01; ^**^P < 0.02; ns, not significant (one-way ANOVA with Tukey’s post hoc test).

We also analyzed the formation of the postoptic commissure in control and mutant embryos at 36 hpf (Fig. 3). In control embryos, the cross-sectional area of the postoptic commissure was 164.8 ± 12.4 μm^2^ (*n* = 11 embryos) (Fig. 3a,h), similar to that of the *rab33a* single mutants and the *rab33ba* single mutants (Fig. 3a–c,h). However, the cross-sectional area of the postoptic commissure was significantly reduced in embryos carrying the *rab33a*;*rab33ba* double mutation (Fig. 3d,h). Furthermore, injection of *rab33a* and *rab33ba* mRNAs, single *rab33a* mRNA or single *rab33ba* mRNA into the double mutant embryos rescued the reduced cross-sectional area of the postoptic commissure (Fig. 3e–h). The body length, head size and distance between eyes were similar among the wild-type, *rab33a* single mutant, *rab33ba* single mutant and *rab33a*;*rab33ba* double mutant fish (Supplementary Fig. S6), thus ruling out the possibility that developmental delays in the double mutant fish results in dysgenesis of the anterior and postoptic commissures. Together, these data indicate that Rab33a and Rab33ba mediate the formation of the anterior and postoptic commissures.

### Rab33a and Rab33ba mediate the outgrowth of anterior commissural axons

**Figure 4:**
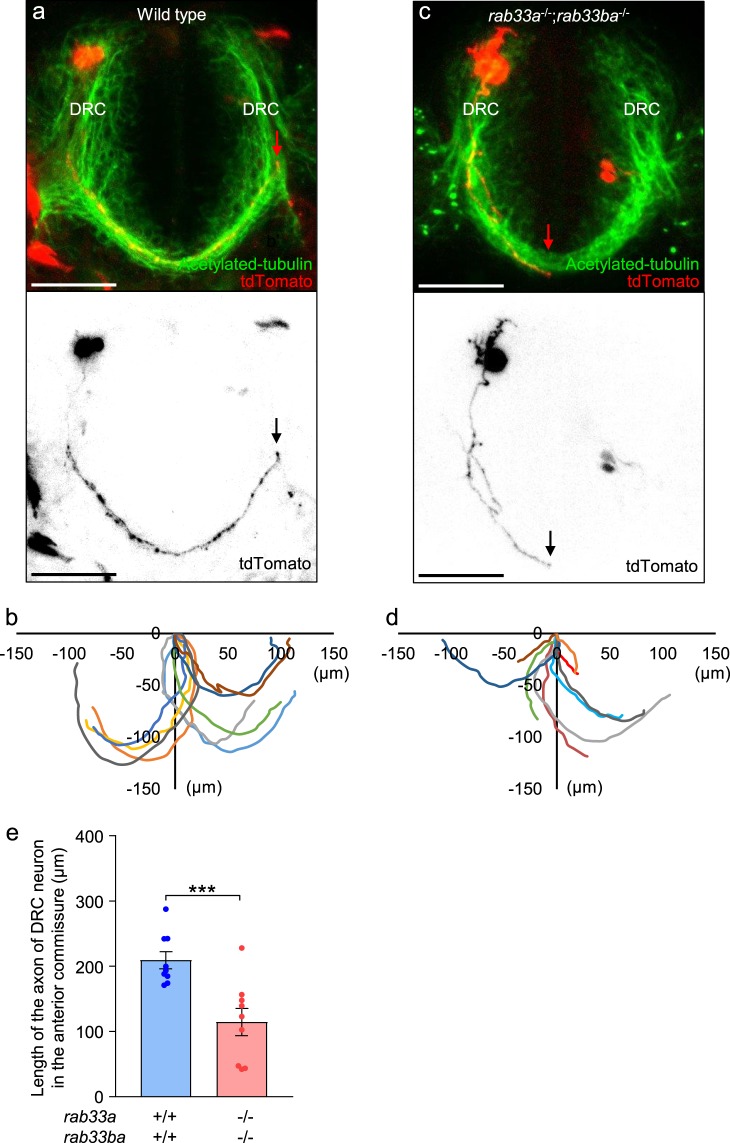
The *rab33a*;*rab33ba* double mutation inhibits axonal extension in the anterior commissure. (**a**,**c**) Representative dorsal views (upper panels) of the anterior commissures immunolabeled with anti-acetylated tubulin antibody and individual DRC neurons expressing tdTomato in wild-type control (**a**) and *rab33a*^−*/*−^;*rab33ba*^−*/*−^ double mutant (**c**) embryos at 36 hpf. The lower panels show only the tdTomato-labeled neurons in the upper panels. The tips of the axons are indicated by arrows. Scale bars: 50 µm. (**b**,**d**) The trajectories of individual axons of the tdTomato-labeled DRC neurons in wild-type control (**b**) and *rab33a*^−*/*−^;*rab33ba*^−*/*−^ double mutant (**e**) embryos at 36 hpf. The cell body positions are normalized at (x = 0 μm, y = 0 μm). (**e**) The length of the axon of DRC neurons in the anterior commissure obtained from the data analyses in (**b**,**d**). Axons of wild-type control (n = 9) and *rab33a*^−*/*−^;*rab33ba*^*−/*−^ double mutant (n = 9) neurons were analyzed at 36 hpf. Data are expressed as mean ± SEM; ^***^P < 0.01 (unpaired Student’s *t* test).

Dysgenesis of the anterior and postoptic commissures in the *rab33a*;*rab33ba* double mutant fish raises the possibility that these molecules promote outgrowth of the forebrain commissure axons cooperatively. Zhang *et al*.^29^ reported a method to trace the projection of individual axons in the anterior commissure using the Gal4/UAS system. To analyze the axonal projection from DRC neurons in the anterior commissure, we coinjected a plasmid carrying *emx3*:Gal4FF and a plasmid carrying UAS:tdTomato into fertilized eggs, as described previously^29^. Under the telencephalon-specific enhancer *emx3*, Gal4FF activates the expression of tdTomato in telencephalic neurons. Figure 4a shows a single tdTomato-labeled DRC neuron in a wild-type 36 hpf embryo. At this stage, most of the labeled axons in the anterior commissure projected to the contralateral DRC (Fig. 4b), The mean length of axons in the anterior commissure was 209.2 ± 13.1 (*n* = 9 cells) (Fig. 4e). On the other hand, the mean axon length in the *rab33a*;*rab33ba* double mutants was 114.5 ± 20.9 (*n* = 9 cells), which is significantly shorter than that observed in control embryos (Fig. 4c–e). These results indicate that Rab33a and Rab33ba are involved in the outgrowth of the anterior commissural axons.

## Discussion

In this study, we showed that *rab33a* and *rab33ba* are expressed in the telencephalic DRC and diencephalic VRC, from which neurons project axons to the anterior and postoptic commissures, respectively. The *rab33a*;*rab33ba* double mutant fish displayed dysgenesis of the anterior and postoptic commissures. Furthermore, the *rab33a*;*rab33ba* double mutation inhibited axonal extension in the anterior commissure. These results suggest that Rab33a and Rab33ba promote the outgrowth of forebrain commissural axons and the formation of forebrain commissures in the developing zebrafish brain. To our knowledge, this is the first report that describes the role of Rab33a in mediating axon outgrowth *in vivo*. In addition, our data provide the first evidence that zebrafish Rab33ba, an ortholog of mammalian Rab33b, is involved in axon outgrowth.

Recent studies reported mutations of *RAB33B* in human patients with Smith–McCort dysplasia^23–25^. Smith-McCort dysplasia is a skeletal dysplasia characterized by a short neck and short trunk dwarfism with a barrel-shaped chest and rhizomelic limb shortening. In the present study, we did not observe these severe phenotypes in the body of the *rab33a*;*rab33ba* double mutant fish. This appears to be consistent with the *in situ* hybridization data, which showed that the bodies of zebrafish embryos lacked clear signals for *rab33a* and *rab33ba*. As *rab33a* and *rab33ba* are expressed in the hindbrain and other forebrain regions, we do not rule out the possibility that they also mediate axon outgrowth of the neurons located in these regions. Further analyses in the hindbrain and other forebrain regions with suitable markers will be required to detect additional defects.

Concerning the molecular mechanism for Rab33a- and Rab33ba-mediated axon outgrowth, *rab33a* single mutant and *rab33ba* single mutant fish did not show remarkable defects although the *rab33a*;*rab33ba* double mutant fish displayed dysgenesis of the forebrain commissures. In addition, injection of single *rab33a* mRNA or single *rab33ba* mRNA into the double mutant embryos rescued the dysgenesis of the forebrain commissures in the *rab33a*;*rab33ba* double mutant. Possible functional redundancy between Rab33a and Rab33ba suggests that these molecules may promote axonal extension thorough a similar mechanism. Our previous study reported that Rab33a in cultured rat hippocampal neurons promotes axonal extension by mediating anterograde axonal transport of post-Golgi vesicles and their concomitant exocytosis at the growth cone^14^. Thus, it is likely that zebrafish Rab33a promotes extension of the forebrain commissural axons by mediating axonal transport of post-Golgi vesicles. In addition, since mammalian Rab33b is also localized in the Golgi apparatus^20–22^, zebrafish Rab33ba may promote axon outgrowth in a similar manner. At present, the effectors of Rab33a that mediate the axonal transport of the post-Golgi vesicles are unknown. The detailed molecular mechanisms how Rab33a and Rab33ba promote axon outgrowth remain for future analyses.

## Methods

### Zebrafish husbandry

All relevant aspects of the experimental procedures were approved by the Institutional Animal Care and Use Committee of Nara Institute of Science and Technology (reference No. 1321 and 1811) and were performed in accordance with relevant guidelines and regulations. Embryos were obtained from wild-type and mutant fish and were raised at 28.5 °C as described previously^32^.

### RT-PCR and cDNA cloning

Total RNA was purified from zebrafish embryos using TRIzol reagent (Invitrogen), according to the manufacturer’s instructions. The RNA sample was used to synthesize cDNA using MLV RT (H-) Point Mutant (Promega) with primer AP (Supplementary Table S1) for reverse transcription. Specific cDNAs were PCR-amplified using the following primers: *rab33a*-h and *rab33a*-t for *rab33a*, and *rab33ba*-h and *rab33ba*-t for *rab33ba*. We used EF1a-f and EF1a-r primers as a positive control. The primers used are listed in Supplementary Table S1. The cDNAs were cloned into pGEM-T and sequenced using an ABI PRISM 3130 (Applied Biosystems).

### DNA electrophoresis

The DNA electrophoresis in Fig. 1a and Supplementary Figs S4c, S5c and S8c–g was performed using agarose gels. The images in Fig. 1a and Supplementary Fig. S8c–g were cropped from full-length gel images in Supplementary Figs S7 and S9, respectively.

### Whole-mount *in situ* hybridization

Whole-mount *in situ* hybridization was performed as described previously^33^. The following plasmids were constructed for synthesizing *in situ* probes. For constructing pCRII-rab33a, *rab33a* was PCR-amplified using the primers rab33a-h and rab33a-t and cloned into pCRII-TOPO (Invitrogen). For constructing pCRII-rab33ba, *rab33ba* in pGEM-T was subcloned into pCRII-TOPO. Plasmids pCRII-rab33a and pCRII-rab33ba were digested with *Not*I, and antisense probes of *rab33a* and *rab33ba* were synthesized using SP6 RNA polymerase (Roche). Plasmids pCRII-rab33a and pCRII-rab33ba were digested with *Bam*HI, and sense probes of *rab33a* and *rab33ba* were synthesized using T7 RNA polymerase (Roche). All *in situ* probes were synthesized from cDNAs using the DIG RNA labeling kit (Roche), according to the manufacturer’s instructions. Images were acquired using a Leica MZFL III and processed using Adobe Photoshop Elements 12 and Fiji^34^.

### Generation of zebrafish *rab33a* and *rab33ba* mutants

Zebrafish mutants of *rab33a* and *rab33ba* were generated using the CRISPR/Cas9 system^35^. Vectors for customized guide RNAs (gRNAs) were constructed as described previously^35^. Plasmid pT7-rab33a was constructed by cloning the two annealed oligonucleotides rab33a-f-ex1 and rab33a-r-ex1. pT7-rab33ba was constructed by cloning the two annealed oligonucleotides rab33ba-f-ex1 and rab33ba-r-ex1. To generate *rab33a* mutants, the gRNAs and Cas9 mRNA were synthesized and injected into wild-type fertilized eggs as described previously^35^. The injected embryos were raised and crossed with wild-type zebrafish. To generate *rab33ba* mutants, we injected *rab33ba* gRNA and Cas9 mRNA into fertilized eggs from the *rab33a* heterozygous mutant fish. The injected fish were raised and crossed with wild-type fish. To identify *rab33a* and *rab33ba* mutations, a T7EI assay was performed as described previously^35^. We used rab33a-ex1-5′ and rab33a-ex1–3′ primers for *rab33a*, and rab33ba-ex1–5′ and rab33ba-ex1–3′ primers for *rab33ba* (Supplementary Table S1). The PCR products were sequenced using an ABI PRISM3130 (Applied Biosystems). We confirmed the mutations of *rab33a* and *rab33ba* by genotyping analyses (Supplementary Figs S4 and S5). In addition, RT-PCR analyses confirmed the absence of the wild-type *rab33a* transcript in *rab33a* single mutant or *rab33a*;*rab33ba* double mutant embryos (Supplementary Fig. S8d). Mutant *rab33ba* transcript expression was detected in *rab33ba* single mutant and *rab33a*;*rab33ba* double mutant embryos, but not in wild-type or *rab33a* single mutant embryos (Supplementary Fig. S8f). We used rab33a-WT-5′ and rab33a-t primers to detect wild-type *rab33a* transcript, and rab33ba-MU-5′ and rab33ba-t primers to detect the mutant *rab33ba* transcript (Supplementary Table S1).

### Genotyping

T7EI-mediated genotyping was performed using the primers rab33a-ex1–5′ and rab33a-ex1–3′ to identify *rab33a* mutations, and rab33ba-ex1–5′ and rab33ba-ex1–3 to identify *rab33ba* mutations. The genotyping was performed using two different T7EI assays. The first T7EI assay was performed as described previously^35^. In the second T7EI assay, PCR products obtained from samples were mixed with those from the wild type before denaturation at 94 °C for 5 min, annealing at room temperature and digestion of the annealed products with T7EI. The first T7EI assay distinguished heterozygous fish from wild-type and homozygous fish. The second T7EI assay distinguished between homozygous and wild-type fish.

### Microinjection

Microinjection was performed as described previously^35^. Fertilized eggs were injected with gRNAs (50 pg/embryo) and Cas9 mRNA (300 pg/embryos). In rescue experiments, fertilized eggs were injected with *rab33a* mRNA (25 pg/embryo) and *rab33ba* mRNA (25 pg/embryo).

### Whole-mount immunohistochemistry

Whole-mount immunohistochemistry was performed as described previously^36^, with slight modifications. Embryos were fixed in 4% formaldehyde (PFA) overnight at 4 °C and washed and blocked with blocking buffer (0.5% Triton-X, 4% normal goat serum, and 0.1% BSA in phosphate buffer) for 2 h at room temperature. The embryos were incubated with anti-acetylated tubulin antibody (Sigma) (1:1000) to label axonal tracts, followed by incubation with Alexa Fluor 488-conjugated anti-mouse IgG (Molecular Probes) (1:500) overnight at 4 °C.

### Measurement of head size and distance between eyes

The head size and distance between eyes of the control and mutant fish were measured as described previously^37^.

### Microscopy

Embryos were embedded in 1% low melting point agarose (Invitrogen). To take confocal images of the postoptic commissure, we removed the yolks from embryos with forceps. Images were captured with a Zeiss LSM710 and the cross-sections of the midline of the forebrain commissures were constructed using Imaris 8.0 and Fiji^34^. The cross-sectional areas were measured using Fiji.

### Genetic single-cell labeling

A mixture of two plasmids emx3:Gal4FF and UAS:tdTomato (10 ng/μl each) was injected into one-cell-stage embryos, as described previously^29^. The injected embryos expressing tdTomato were screened by a fluorescent dissection microscopy at 24 hpf and fixed at 36 hpf. The embryos were immunostained with anti-acetylated tubulin antibody and observed by a Zeiss LSM710. Axon length was measured using Fiji.

### Statistical analysis

Results are expressed as mean ± standard error (SEM). Statistical analyses were performed with GraphPad Prism 7. Statistical significance was determined by the unpaired Student’s *t* test. For multiple comparisons, we used one-way ANOVA with Tukey’s post hoc test.

## Supplementary information

Supplementary information

Movie 1

## References

[1] Pfenninger KH (2009). Plasma membrane expansion: a neuron’s Herculean task. Nat. Rev. Neurosci..

[2] Bray D (1970). Surface movements during the growth of single explanted neurons. Proc. Natl. Acad. Sci. USA.

[3] Lockerbie RO, Miller VE, Pfenninger KH (1991). Regulated plasmalemmal expansion in nerve growth cones. J. Cell. Biol..

[4] Craig AM, Wyborski RJ, Banker G (1995). Preferential addition of newly synthesized membrane protein at axonal growth cones. Nature.

[5] Futerman AH (1996). & Banker, G. A. The economics of neurite outgrowth–the addition of new membrane to growing axons. Trends. Neurosci..

[6] Pfeffer SR (2001). Rab GTPases: specifying and deciphering organelle identity and function. Trends Cell Biol..

[7] Zerial M, McBride H (2001). Rab proteins as membrane organizers. Nat. Rev. Mol. Cell Biol..

[8] Fukuda M (2008). Regulation of secretory vesicle traffic by Rab small GTPases. Cell. Mol. Life Sci..

[9] Stenmark H (2009). Rab GTPases as coordinators of vesicle traffic. Nat. Rev. Mol. Cell Biol..

[10] Zhen Y, Stenmark H (2015). Cellular functions of Rab GTPases at a glance. J. Cell Sci..

[11] Koda T, Kakinuma M (1993). Molecular cloning of a cDNA encoding a novel small GTP-binding protein. FEBS Lett..

[12] Cheng E (2006). Rab33A: characterization, expression, and suppression by epigenetic modification. J. Invest. Dermatol..

[13] Lee MS (2006). Selection of neural differentiation-specific genes by comparing profiles of random differentiation. Stem Cells.

[14] Nakazawa H (2012). Rab33a mediates anterograde vesicular transport for membrane exocytosis and axon outgrowth. J. Neurosci..

[15] Imai A, Tsujimura M, Yoshie S, Fukuda M (2015). The small GTPase Rab33A participates in regulation of amylase release from parotid acinar cells. Biochem. Biophys. Res. Commun..

[16] Ishibashi K, Uemura T, Waguri S, Fukuda M (2012). Atg16L1, an essential factor for canonical autophagy, participates in hormone secretion from PC12 cells independently of autophagic activity. Mol. Biol. Cell.

[17] Zografou S (2012). A complete Rab screening reveals novel insights in Weibel-Palade body exocytosis. J. Cell Sci..

[18] Fukuda M, Kobayashi H, Ishibashi K, Ohbayashi N (2011). Genome-wide investigation of the Rab binding activity of RUN domains: development of a novel tool that specifically traps GTP-Rab35. Cell Struct. Funct..

[19] Mori T, Wada T, Suzuki T, Kubota Y, Inagaki N (2007). Singar1, a novel RUN domain-containing protein, suppresses formation of surplus axons for neuronal polarity. J. Biol. Chem..

[20] Zheng JY (1998). A novel Rab GTPase, Rab33B, is ubiquitously expressed and localized to the medial Golgi cisternae. J. Cell Sci..

[21] Valsdottir R (2001). Identification of rabaptin-5, rabex-5, and GM130 as putative effectors of rab33b, a regulator of retrograde traffic between the Golgi apparatus and ER. FEBS Lett..

[22] Itoh T (2008). Golgi-resident small GTPase Rab33B interacts with Atg16L and modulates autophagosome formation. Mol. Biol. Cell.

[23] Alshammari MJ, Al-Otaibi L, Alkuraya FS (2012). Mutation in RAB33B, which encodes a regulator of retrograde Golgi transport, defines a second Dyggve–Melchior–Clausen locus. J. Med. Genet..

[24] Dupuis N (2013). A novel RAB33B mutation in Smith-McCort dysplasia. Hum. Mutat..

[25] Salian S (2017). Additional three patients with Smith-McCort dysplasia due to novel RAB33B mutations. Am. J. Med. Genet. A.

[26] Hall TE, Martel N, Lo HP, Xiong Z, Parton RG (2017). A plasmid library of full-length zebrafish rab proteins for *in vivo* cell biology. Cell. Logist..

[27] Chitnis AB, Kuwada JY (1990). Axonogenesis in the brain of zebrafish embryos. J. Neurosci..

[28] Ross LS, Parrett T, Easter SS (1992). Axonogenesis and morphogenesis in the embryonic zebrafish brain. J. Neurosci..

[29] Zhang C, Gao J, Zhang H, Sun L, Peng G (2012). Robo2-Slit and Dcc-Netrin1 coordinate neuron axonal pathfinding within the embryonic axon tracts. J. Neurosci..

[30] Barresi MJ, Hutson LD, Chien CB, Karlstrom RO (2005). Hedgehog regulated Slit expression determines commissure and glial cell position in the zebrafish forebrain. Development.

[31] Wilson SW, Ross LS, Parrett T, Easter SS (1990). The development of a simple scaffold of axon tracts in the brain of the embryonic zebrafish, Brachydanio rerio. Development.

[32] Westerfield, M. The zebrafish book. *University of Oregon Press* (2007).

[33] Schulte-Merker, S. Looking at embryos. In zebrafish: a practical approach. *Oxford Univ Press*, 39–58 (2002).

[34] Schindelin J (2012). Fiji: an open-source platform for biological-image analysis. Nat. Methods.

[35] Jao LE, Wente SR, Chen W (2013). Efficient multiplex biallelic zebrafish genome editing using a CRISPR nuclease system. Proc. Natl. Acad. Sci. USA.

[36] Karlstrom RO (1996). Zebrafish mutations affecting retinotectal axon pathfinding. Development.

[37] Loviglio MN (2017). The immune signaling adaptor LAT contributes to the neuroanatomical phenotype of 16p11.2 BP2-BP3 CNVs. Am. J. Hum. Genet..

