# Supplementary material for: Rab33a and Rab33ba mediate the outgrowth of forebrain commissural axons in the zebrafish brain
Source: Sci Rep. 2019 Feb 12;9:1799. doi: 10.1038/s41598-018-38468-5 (PMC6372587; doi:10.1038/s41598-018-38468-5)
Supplement: Supplementary file 1 — Supplementary information [file 41598_2018_38468_MOESM1_ESM.docx]

**Supplementary information**

**Rab33a and Rab33ba mediate the outgrowth of forebrain commissural axons in the zebrafish brain**

Liguo Huang, Akihiro Urasaki & Naoyuki Inagaki

**
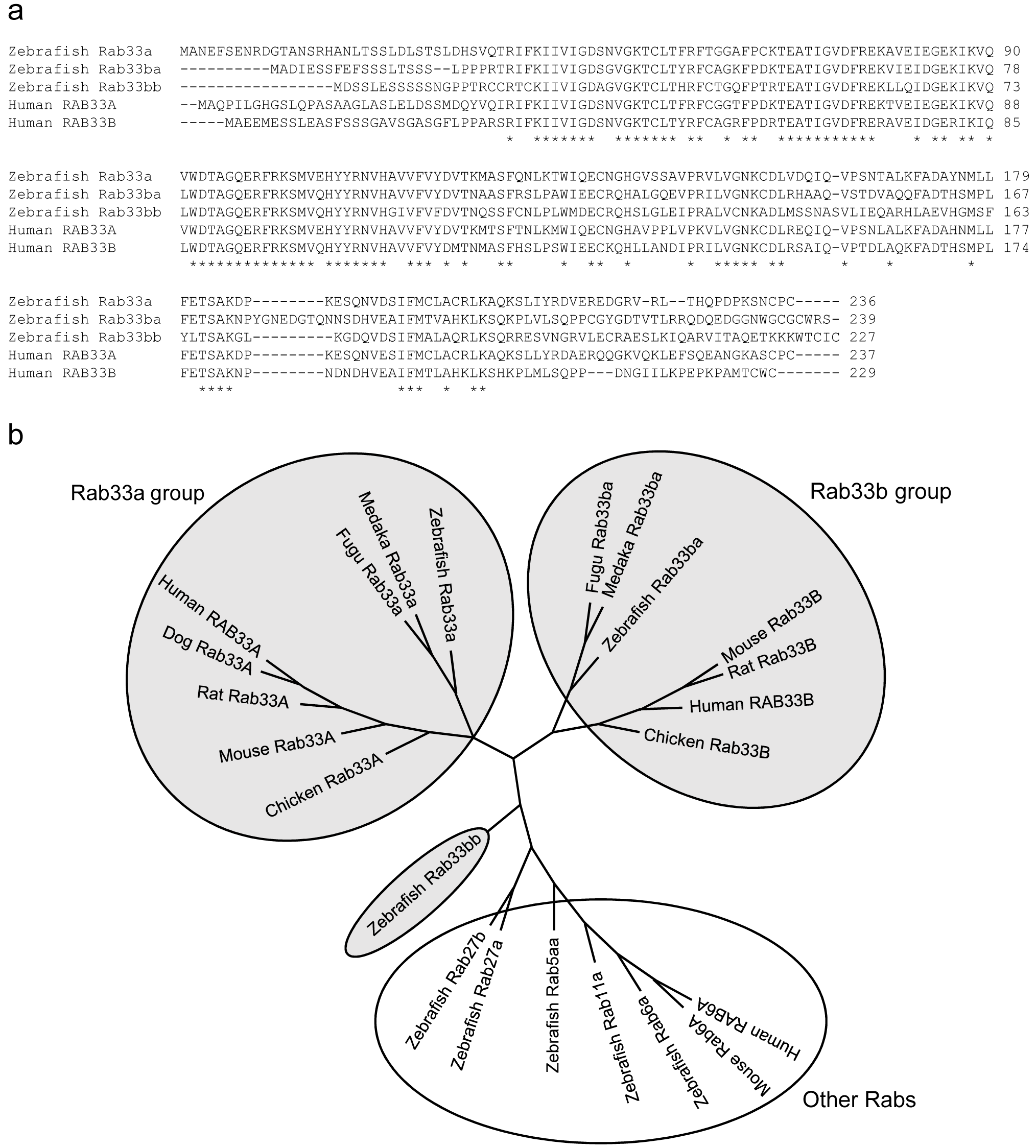
**

**Supplementary Figure S1. Multiple sequence alignment and phylogenetic tree analyses of Rab33 proteins.**

(a) Amino acid sequences of zebrafish Rab33a, Rab33ba and Rab33bb aligned with human RAB33A and RAB33B. Identical residues are indicated by asterisks. (b) Phylogenetic tree of vertebrate Rab33 and other Rabs. Accession numbers of the protein sequences used in the phylogenetic analysis are listed in Supplementary Table S2.


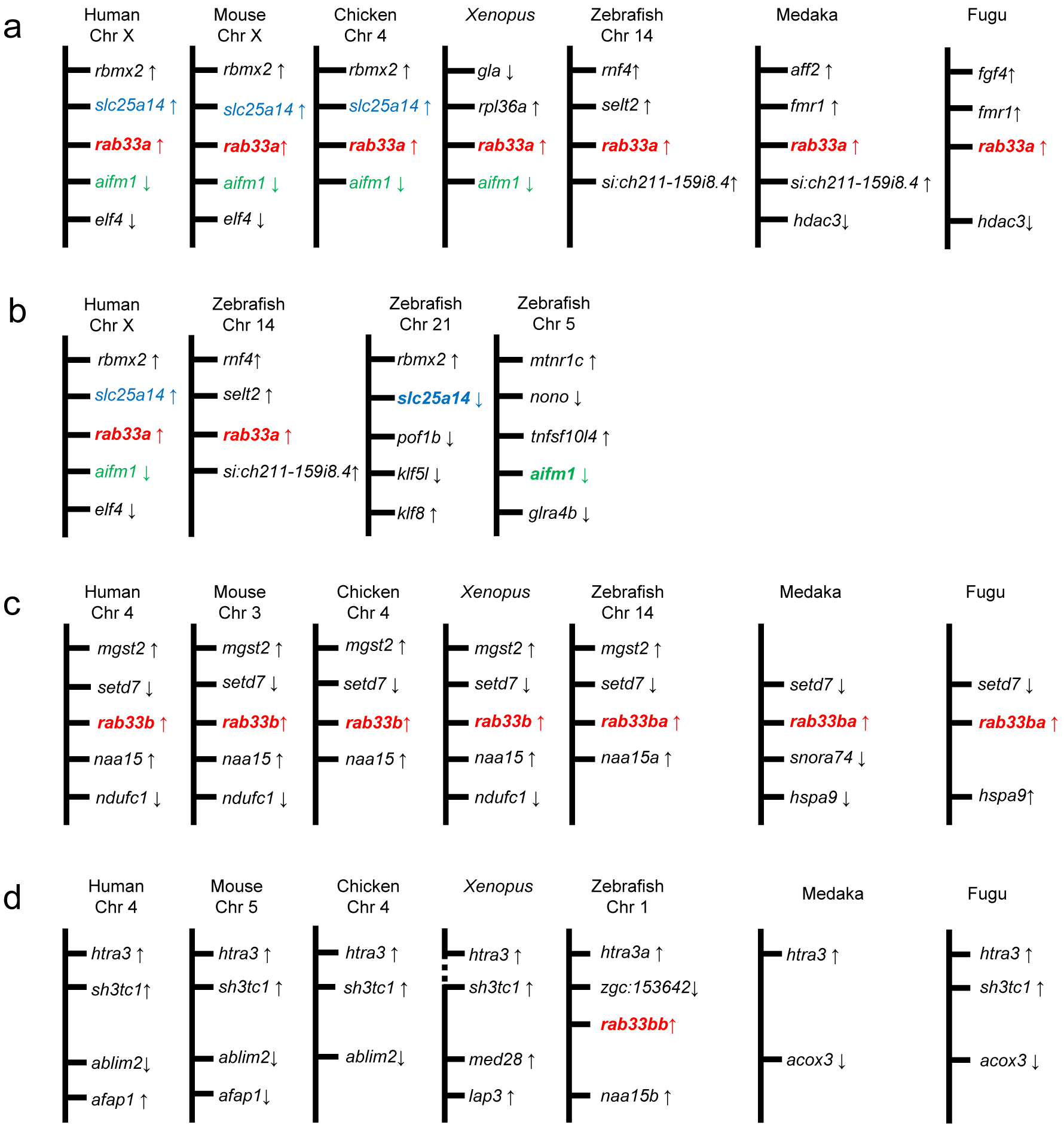


**Supplementary Figure S2. Synteny analyses of Rab33 genes.**

(a, c and d) Synteny analyses of *rab33a* (a), *rab33b* and *rab33ba* (c), and *rab33bb* (d) genes in human, mouse, chicken, *Xenopus*, zebrafish, medaka and fugu genome assemblies. (b) Synteny analyses of human *rab33a*, zebrafish *rab33a*, zebrafish *slc25a14* and zebrafish *aifm1*. Arrows indicate the translational orientation of genes.


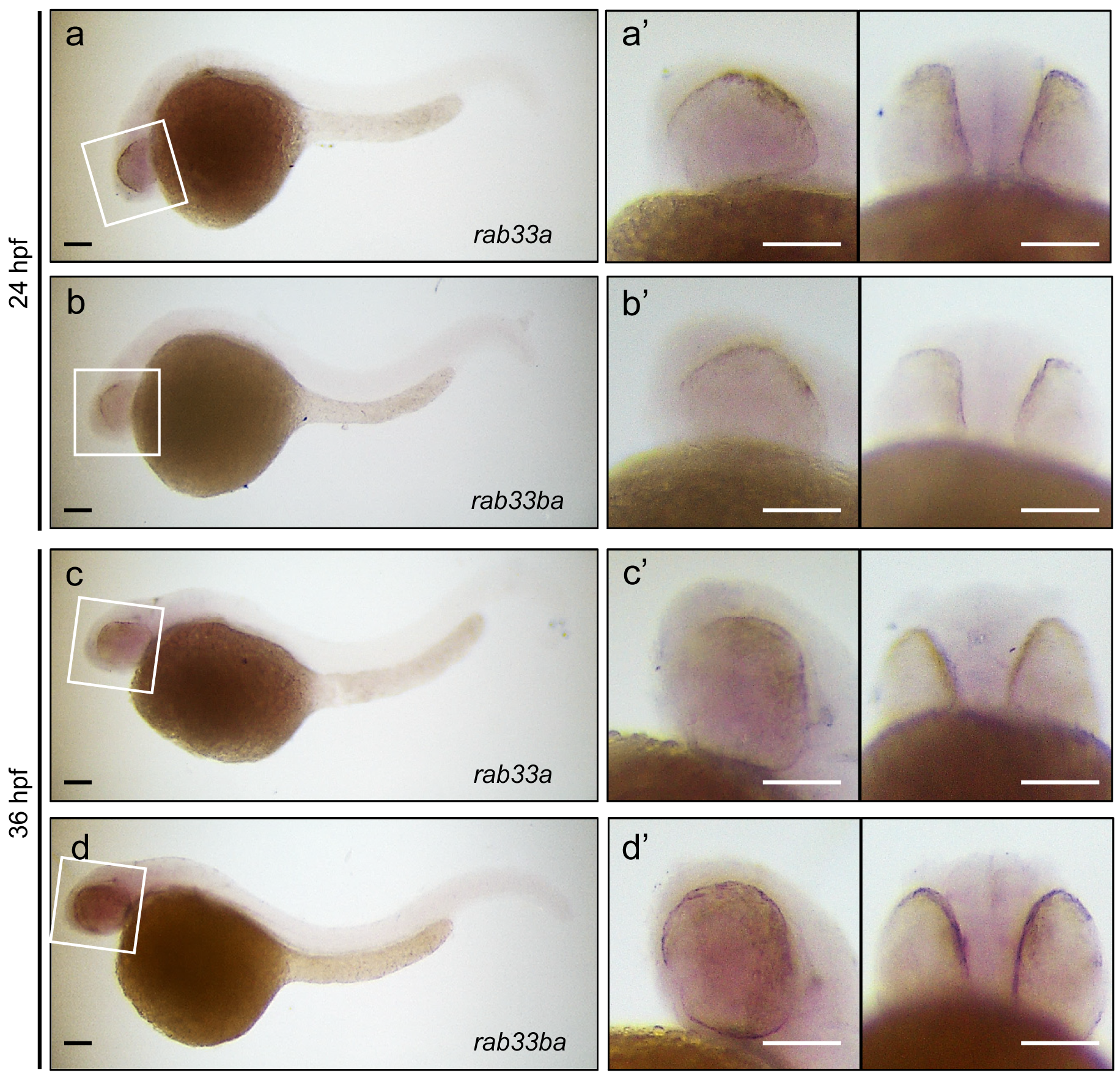


**Supplementary Figure S3. Negative control data for the whole-mount *in situ* hybridization of *rab33a* and *rab33ba* in Figure 1b-e.**

Whole-mount *in situ* hybridizations of *rab33a* (a, c) and *rab33ba* (b, d) at 24 hpf (a, b) and at 36 hpf (c, d) were performed using the sense probes as controls; (a’-d’) show the enlarged lateral (left) and ventral (right) views of the areas indicated by the rectangles. Scale bars: 100 μm.

**
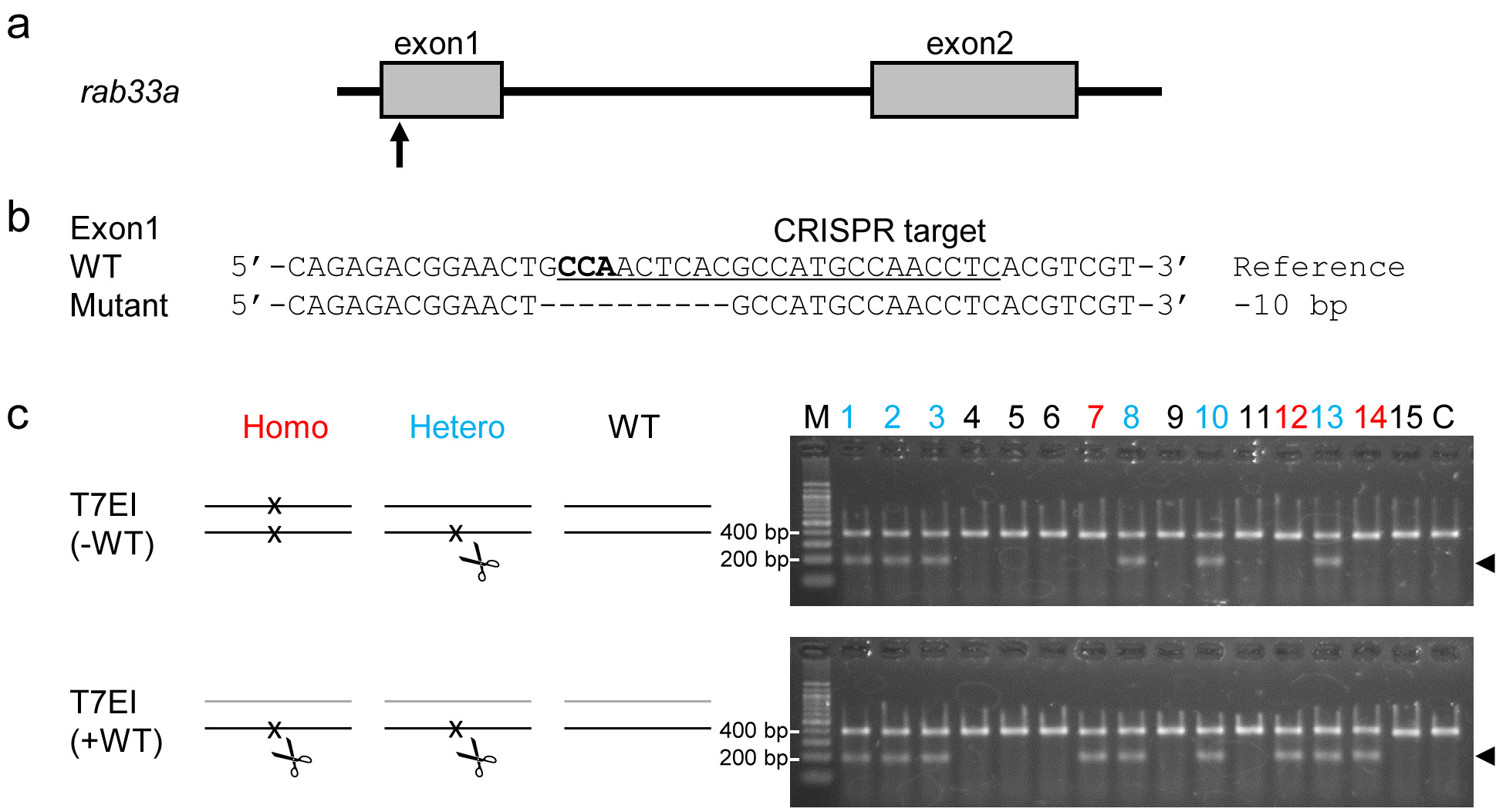
**

**Supplementary Figure S4. Generation of a *rab33a* mutant using the CRISPR/Cas9 system.**

(a) Schematic representation of the genomic structure of the *rab33a* gene. The arrow indicates the position of the CRISPR target in *rab33a*. (b) DNA sequences of the *rab33a* CRISPR target in the wild type and *rab33a* mutant. The underline indicates the sequence of the *rab33a* CRISPR target site. Bold letters indicate protospacer adjacent motif (PAM) sequences. The *rab33a* mutant fish has a mutation in exon1. (c) T7EI-based *rab33a* genotyping. PCR reactions were performed using *rab33a*-specific primers. PCR products were denatured and reannealed without wild-type PCR products (–WT) or with wild-type PCR products (+WT). The annealed double-strand DNAs were treated with T7EI and analyzed by electrophoresis in 2.5% agarose gels. Arrowheads indicate T7EI-digested bands. The different colors of lane numbers indicate different genetic backgrounds: homozygous (red), heterozygous (blue) and wild type (black). Lane M, DNA marker; lane C, PCR products obtained using wild-type genomic DNA template as controls.

**
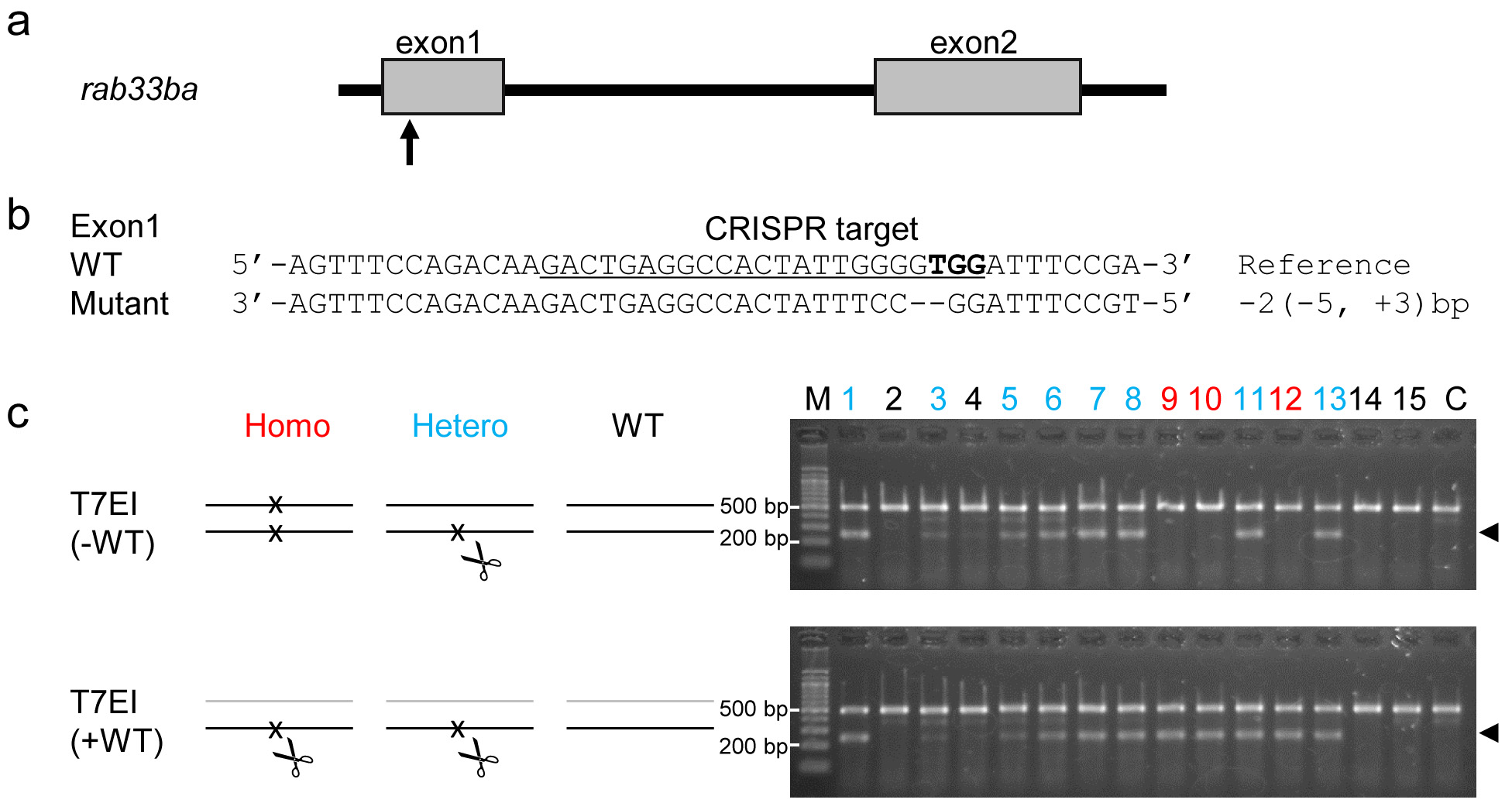
**

**Supplementary Figure S5. Generation of a *rab33ba* mutant using the CRISPR/Cas9 system.**

(a) Schematic representation of the genomic structure of the *rab33ba* gene. The arrow indicates the position of the CRISPR target in *rab33ba*. (b) DNA sequences of *rab33ba* in the wild type and *rab33ba* mutant. The underline indicates the sequence of the CRISPR target site. Bold letters indicate the PAM sequence. The *rab33ba* mutant fish carried a mutation in exon1. (c) T7EI-based *rab33ba* genotyping. PCR reactions were performed using *rab33ba*-specific primers. PCR products were denatured and reannealed without wild-type PCR products (–WT) or with wild-type PCR products (+WT). The annealed double-strand DNAs were treated with T7EI and analyzed by electrophoresis in 2.5% agarose gels. Arrowheads indicate T7EI-digested bands. The different colors of lane numbers indicate different genetic backgrounds: homozygous (red), heterozygous (blue) and wild type (black). Lane M, DNA marker; lane C, PCR products obtained using wild-type genomic DNA template as controls.

**Supplementary Figure S6. Body length and head size of wild-type, *rab33a* single mutant, *rab33ba* single mutant and *rab33a*;*rab33ba* double mutant embryos.**


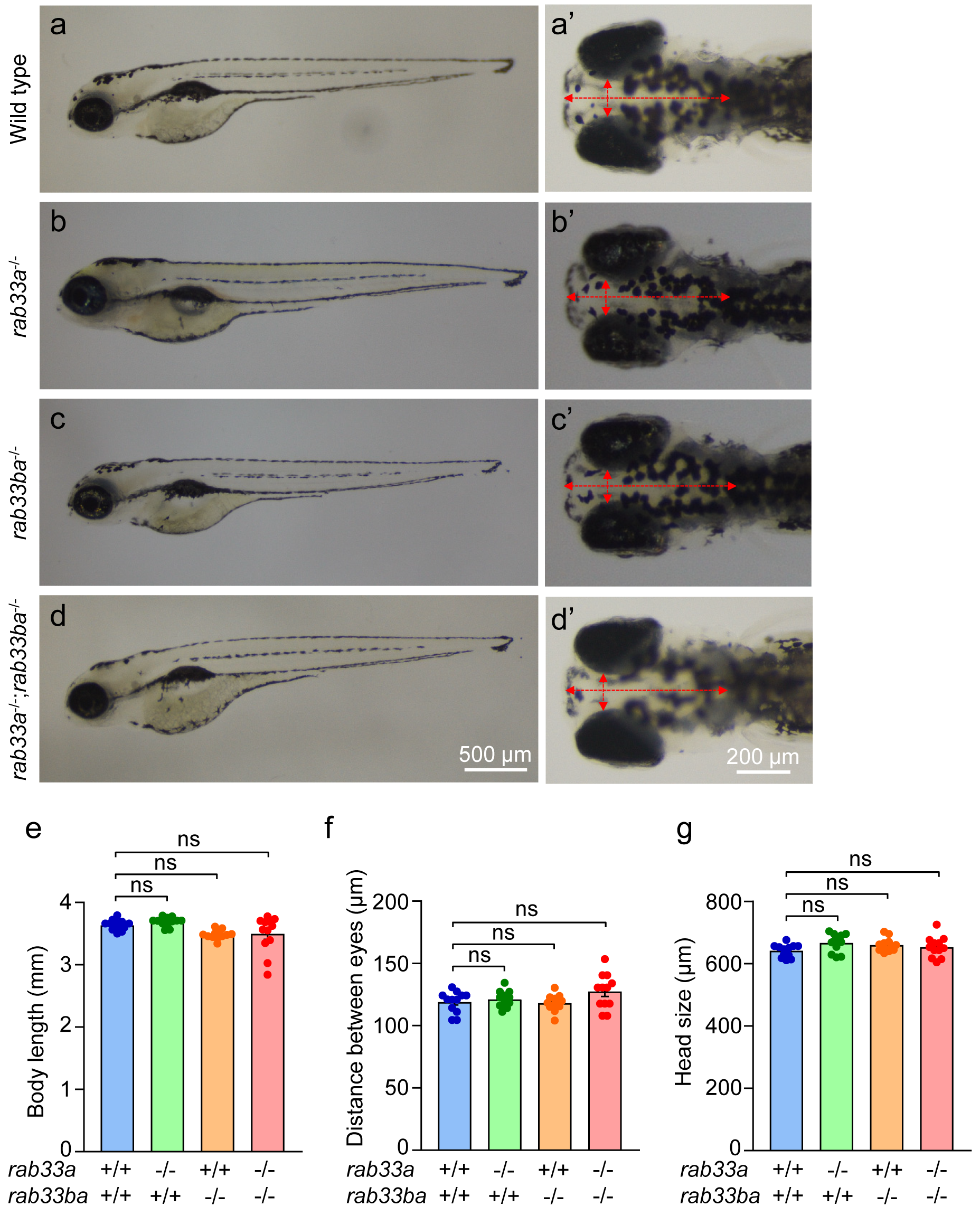


(a-d) Lateral views of the whole body of 100 hpf wild-type (a), *rab33a* single mutant (b), *rab33ba* single mutant (c) and *rab33a;rab33ba* double mutant (d) embryos. Scale bar: 500 µm. (a’–d’) Dorsal views of the head of the embryos in (a-d). Scale bar: 200 µm. (e-g) The body length (e), distance between eyes (f) and head size (g) obtained from the data analyses in (a–d and a’-d’). Wild-type control (n = 12), *rab33a^-/-^* single mutant (n = 12), *rab33ba^-/-^* single mutant (n = 12) and *rab33a^-/-^*;*rab33ba^-/-^* double mutant (n = 12) embryos were analyzed. Data are expressed as mean ± SEM; ns, not significant (one-way ANOVA with Tukey’s post hoc test).

The full-length gel image in Figure 1a *rab33a*

*
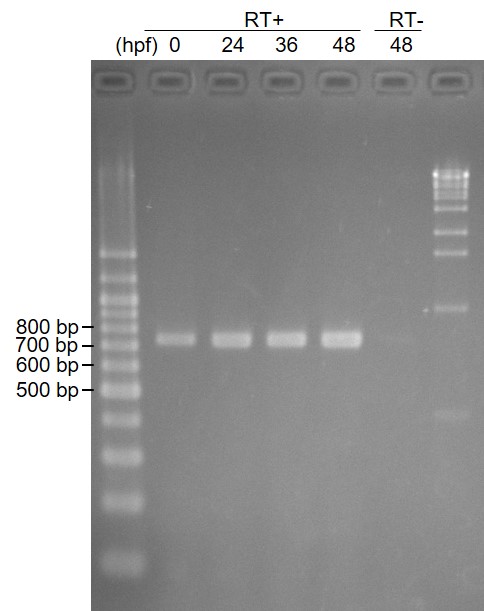
*

The full-length gel image in Figure 1a *rab33ba
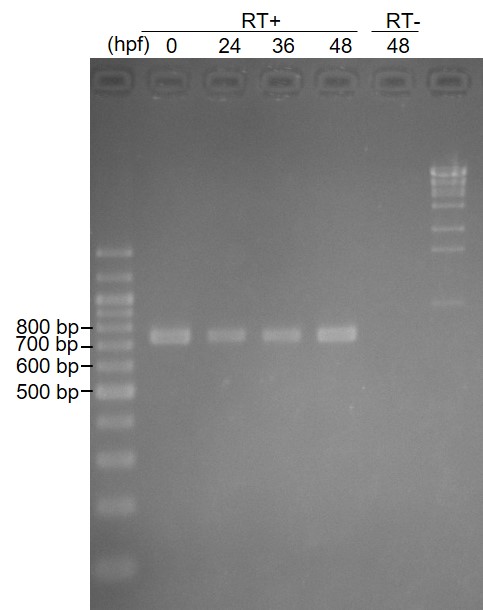
*

The full-length gel image in Figure 1a EF1a

**
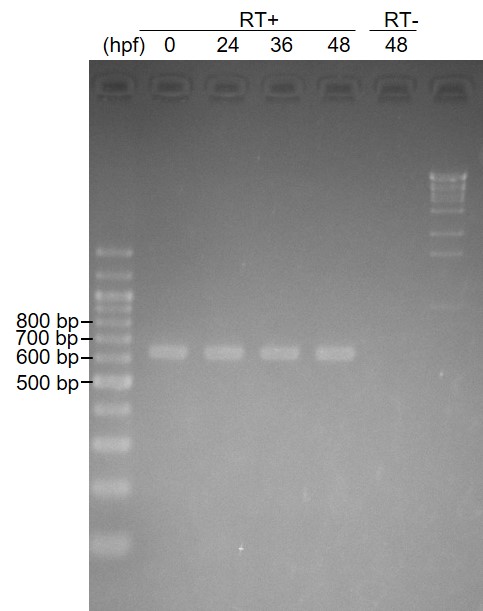
**

**Supplementary Figure S7. Full-length gel images in Figure 1a.**

Full length gel images of *rab33a*, *rab33ba* and EF1a in Figure 1a. All the gel images are raw data without modification.

**Supplementary Figure S8. RT-PCR analyses of *rab33a* and *rab33ba* transcripts.**


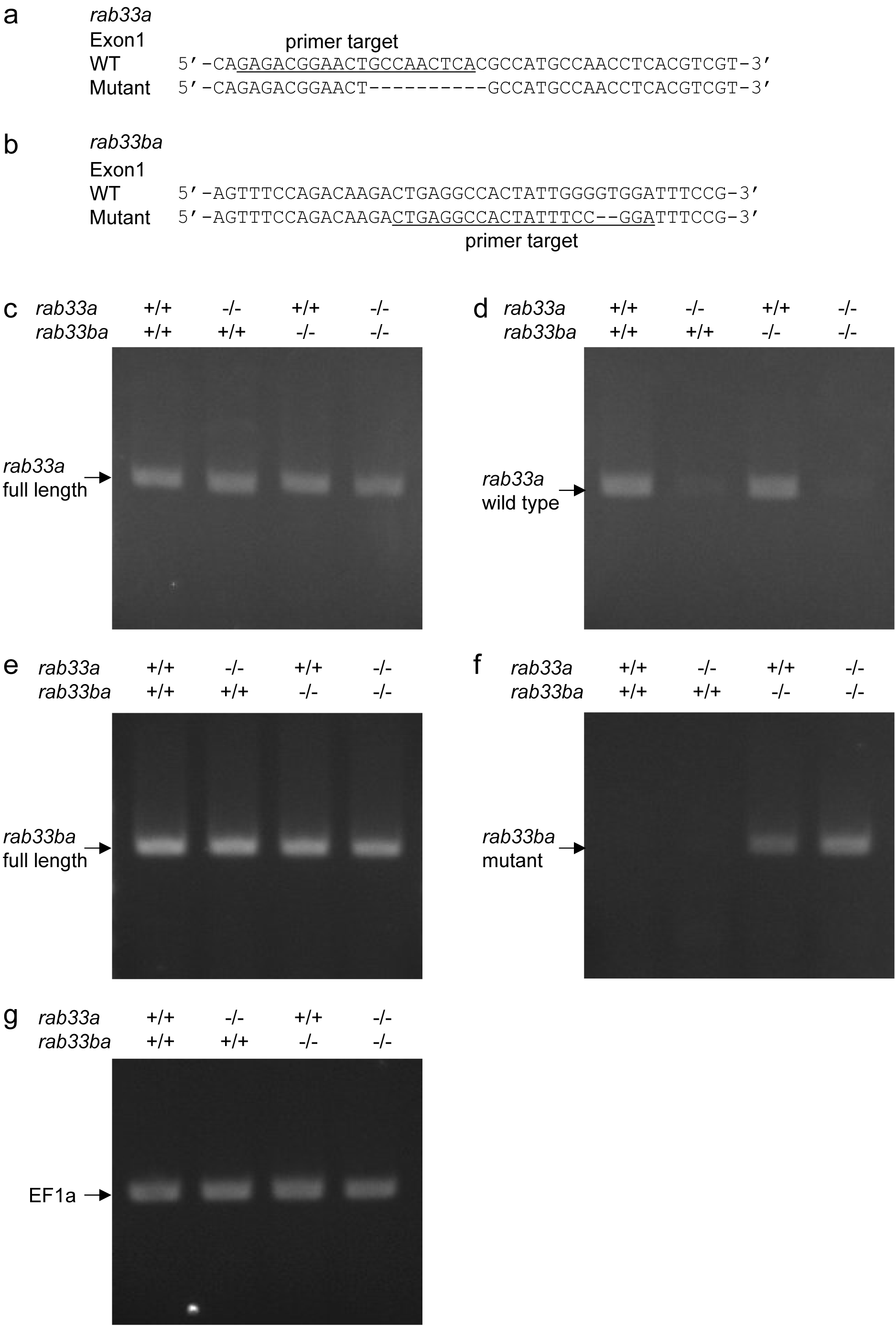


(a) The sequence of *rab33a* wild-type specific primer target. (b) The sequence of *rab33ba* mutant specific primer target. (c-f) The expressions of full length *rab33a* (c), wild-type *rab33a* (d), full length *rab33ba* (e) and mutant *rab33ba* (f) were analyzed in wild-type, *rab33a* single mutant, *rab33ba* single mutant and *rab33a*;*rab33ba* double mutant embryos. (g) Elongation factor 1a (EF1a) was used as a control. RT-PCR products were electrophoresed on 2% agarose gels. The images were cropped from full-length gel images (Supplementary Fig. S9).

The full-length gel image in Supplementary Figure S8
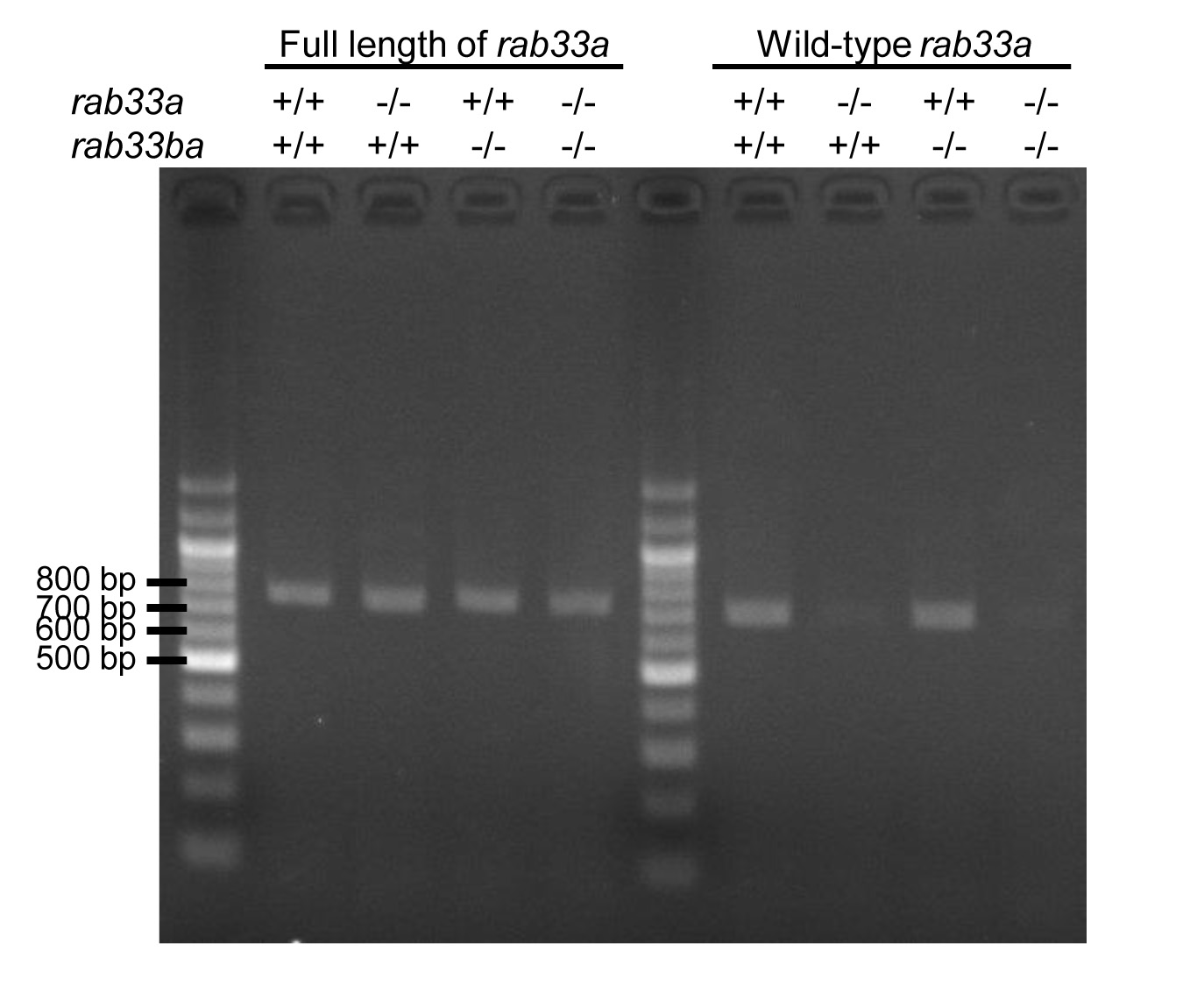
c and d

The full-length gel image in Supplementary Figure S8e and f

**
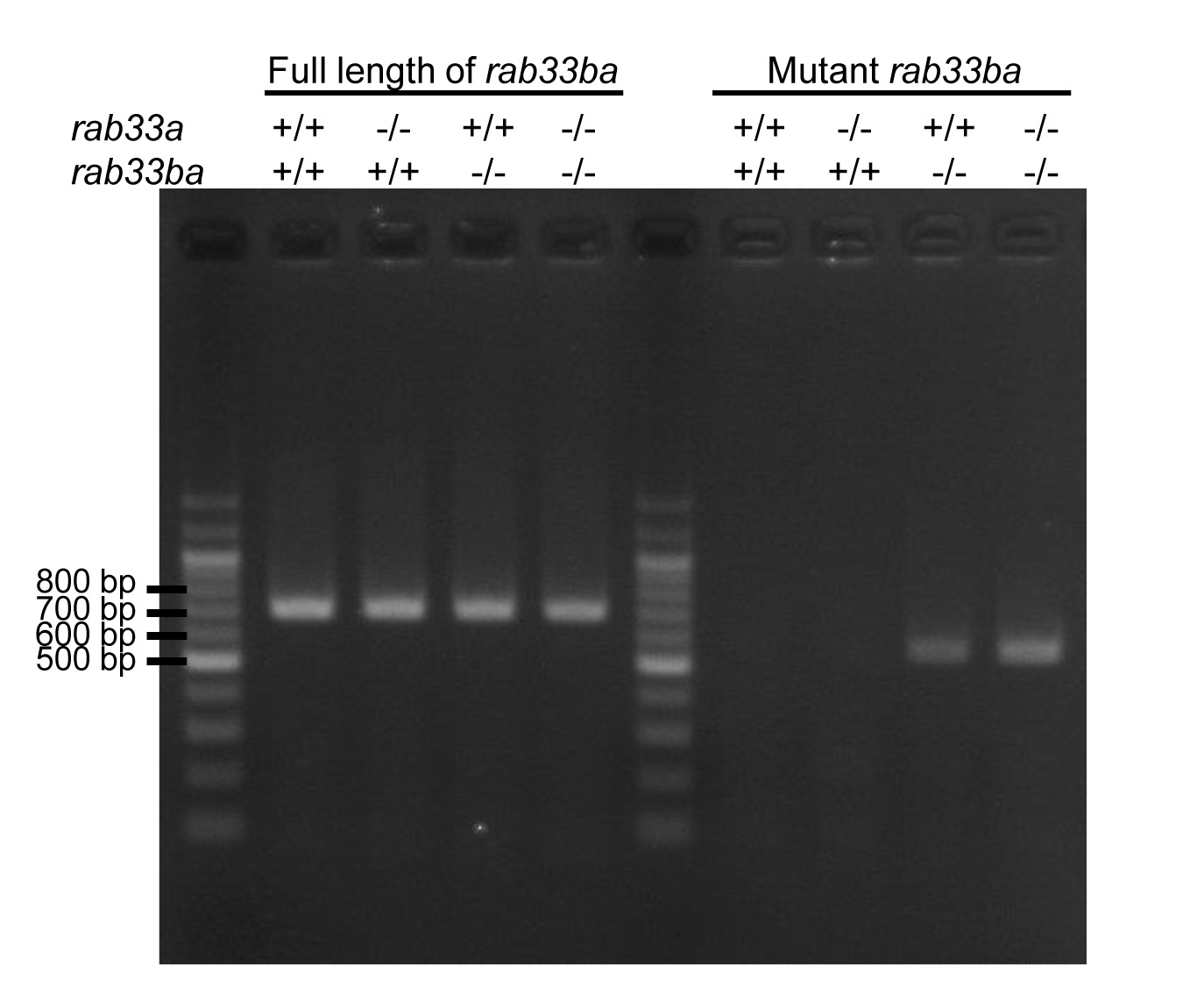
**

The full-length gel image in Supplementary Figure S8g

**
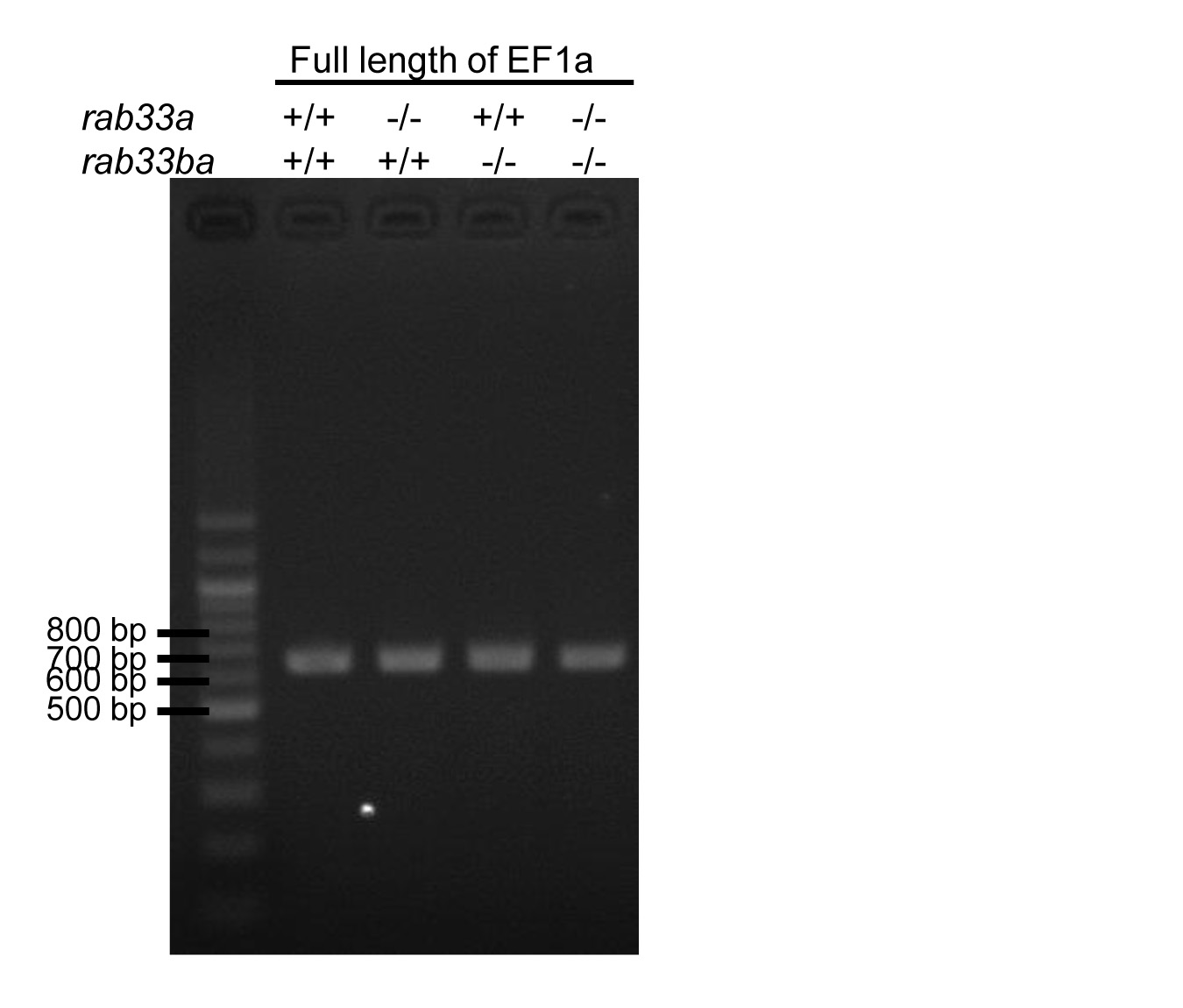
**

**Supplementary Figure S9. Full-length gel images in Supplementary Figure S8.**

All the gel images are raw data without modification.

**Supplementary Table S1. Oligonucleotide list.**

**-------------------------------------------------------------------------------------------------------------------------**

Name Sequence (5’ to 3’)

**-------------------------------------------------------------------------------------------------------------------------**

AP GGCCACGCGTCGACTAGTACTTTTTTTTTTTTTTTTT

EF1a-f AGCGGTACTACTCTTCTTGATGC

EF1a-r TTGTACACATCCTGAAGTGGCA

rab33a-h AAAGGATCCGCCACCATGGCAAATGAATTCTCAGAAAACA

rab33a-t TTTGCGGCCGCTCTAGATCAGCACGGGCAGTTACTCTTGGG

rab33ba-h TTTGGATCCGCCACCATGGCAGATATCGAGTCCTCTTTTGA

rab33ba-t　　　　　　　　 AAAGCGGCCGCTCTAGATTAGCTTCTCCAACAACCGCAGC

rab33a-f-ex1　　　　　　 TAGGGAGGTTGGCATGGCGTGAGT

rab33a-r-ex1 　　　　　 AAACACTCACGCCATGCCAACCTC

rab33ba-f-ex1 TAGGACTGAGGCCACTATTGGGG

rab33ba-r-ex1 AAACCCCCAATAGTGGCCTCAGT

rab33a-ex1-5' CTTAAATCAACTACATCAGTTGGCAAACAC

rab33a-ex1-3' TCCACCGCTTTCTCCCTGAAATCCACGCCG

rab33ba-ex1-5' CGGCATTACTACATTTGCACGGTGTCAGCC

rab33ba-ex1-3' ACGGTCACAGTACAAAAATGAACAAATG

rab33a-WT-5’ GAGACGGAACTGCCAACTCA

rab33ba-MU-5’ CTGAGGCCACTATTTCCGGA

**Supplementary Table S2. Accession numbers of protein sequences used in the phylogenetic analysis.**

**-------------------------------------------------------------------------------------------------------------------------**

Name Accession numbers

**-------------------------------------------------------------------------------------------------------------------------**

*Danio rerio* (zebrafish) rab33a ENSDARG00000057394

*Danio rerio* (zebrafish) rab33ba ENSDART00000074116.5_1

*Danio rerio* (zebrafish) rab33bb Addgene plasmid#80524

*Danio rerio* (zebrafish) rab5aa ENSDART00000034124.8

*Danio rerio* (zebrafish) rab6a ENSDART00000171392.2

*Danio rerio* (zebrafish) rab11a ENSDART00000060766.4

*Danio rerio* (zebrafish) rab27a ENSDART00000165301.2

*Danio rerio* (zebrafish) rab27b ENSDART00000193570.1

*Homo sapiens* (human) RAB33A ENST00000257017.4_1

*Homo sapiens* (human) RAB33B ENST00000305626.5_1

*Homo sapiens* (human) RAB6A ENST00000310653.10

*Rattus norvegicus* (Rat) Rab33a ENSRNOT00000008868.4

*Rattus norvegicus* (Rat) Rab33b ENSRNOT00000017396.5

*Mus musculus* (mouse) Rab33a ENSMUST00000033430.2_1

*Mus musculus* (mouse) Rab33b ENSMUST00000054387.7_1

*Mus musculus* (mouse) Rab6a ENSMUST00000107048.7

*Canis lupus familiaris* (dog) Rab33a ENSCAFT00000029768.2

*Gallus gallus* (chicken) Rab33a ENSGALT00000040577.3

*Gallus gallus* (chicken) Rab33b ENSGALT00000015926.3

*Takifugu rubripes* (fugu) rab33a ENSTRUT00000015022.1_1

*Takifugu rubripes* (fugu) rab33ba ENSTRUT00000017361.1

*Oryzias latipes* (medaka) rab33a ENSORLT00000011342.1_1

*Oryzias latipes* (medaka) rab33ba ENSORLT00000013348.1_1

**Movie 1.** 3D movie of a 36 hpf wild-type zebrafish brain immunolabeled with anti-acetylated tubulin (Fig. 2b).
